# Decreased Level of Nurr1 in Heterozygous Young Adult Mice Leads to Exacerbated Acute and Long-Term Toxicity after Repeated Methamphetamine Exposure

**DOI:** 10.1371/journal.pone.0015193

**Published:** 2010-12-03

**Authors:** Yu Luo, Yun Wang, Serena Y. Kuang, Yung-Hsiao Chiang, Barry Hoffer

**Affiliations:** 1 National Institute on Drug Abuse, Intramural Research Program, Baltimore, Maryland, United States of America; 2 Division of Neurosurgery, Department of Surgery, Taipei Medical University Hospital, Taipei City, Taiwan Authority; 3 Department of Surgery, College of Medicine, Taipei Medical University, Taipei City, Taiwan Authority; Pennsylvania State University, United States of America

## Abstract

The abuse of psychostimulants, such as methamphetamine (METH), is prevalent in young adults and could lead to long-term adaptations in the midbrain dopamine system in abstinent human METH abusers. Nurr1 is a gene that is critical for the survival and maintenance of dopaminergic neurons and has been implicated in dopaminergic neuron related disorders. In this study, we examined the synergistic effects of repeated early exposure to methamphetamine in adolescence and reduction in Nurr1 gene levels. METH binge exposure in adolescence led to greater damage in the nigrostrial dopaminergic system when mice were exposed to METH binge later in life, suggesting a long-term adverse effect on the dopaminergic system. Compared to naïve mice that received METH binge treatment for the first time, mice pretreated with METH in adolescence showed a greater loss of tyrosine hydroxylase (TH) immunoreactivity in striatum, loss of THir fibers in the substantia nigra reticulata (SNr) as well as decreased dopamine transporter (DAT) level and compromised DA clearance in striatum. These effects were further exacerbated in Nurr1 heterozygous mice. Our data suggest that a prolonged adverse effect exists following adolescent METH binge exposure which may lead to greater damage to the dopaminergic system when exposed to repeated METH later in life. Furthermore, our data support that Nurr1 mutations or deficiency could be a potential genetic predisposition which may lead to higher vulnerability in some individuals.

## Introduction

Nurr1 is one of the most important genes for the development and maintenance of dopaminergic (DA-ergic) neurons. Loss of Nurr1 gene during development leads to absence of midbrain DAergic neurons [1]. Nurr1 is expressed throughout the adulthood in mice [2]. It regulates several important genes that are involved in the synthesis and metabolism of DA [3] as well supports the survival of DAergic neurons [4]. A recent study [5] indicated that reduction of Nurr1 function in adulthood leads to a slowly progressive loss of striatal DA and markers for DAergic neurons, supporting its selective roles in the maintenance of DAergic neuronal survival and function. Deficiency in Nurr-1 expression results in a Parkinson's disease (PD)-like phenotype. For example, there were more DAergic neurons lost in the substantia nigra compacta than in the ventral tegmental area when Nurr1 was deleted in maturing DAergic neurons [5]. Nurr1 heterozygous mice, which have decreased Nurr1 mRNA and protein levels, are more vulnerable to injury induced by the DAergic toxin (1-methyl-4-phenyl-1,2,3,6-tetrahydropyridine) MPTP [6]. Furthermore, Nurr1 expression is diminished in neurons with alpha-synuclein inclusions in postmortem PD brain tissue [7]; *Nurr1* mutations and polymorphisms have also been identified in rare cases of PD [7], [8], [9], [10]. Taken together, these data suggest that deficiency in Nurr1 expression may enhance susceptibility to neuronal damage in DAergic neurons, which leads to PD- like symptoms in animals or man.

Methamphetamine (METH) is a commonly abused drug and DAergic neurotoxin. METH causes damage to nigrostriatal DAergic neurons as evidenced by marked decreases in the neostriatal content of DA and activity of tyrosine hydroxylase (TH) [11], [12], [13]. METH selectively injures the neurites of DA neurons, generally without inducing cell death [14]. Administration of METH also enhanced nNOS (neuronal nitric oxide synthase) and 3-nitrotyrosine level in the striatum. These Meth-associated neurodegenerative effects were further potentiated in Nurr1 heterozygous mice [15].

The purpose of this study was to examine the long term effect of repeated methamphetamine exposure in Nurr1 deficient heterozygous mice. Our data suggest that repeated METH binge exposure lead to greater damage in DA neurons and that a deficiency in Nurr1 expression further potentiates METH toxicity.

## Materials and Methods

### Animals and drug administration

The use of animals was conducted under National Institutes Health (NIH) Guidelines using the NIH handbook *Animals in Research* and was approved by the Institutional Animal Care and Use Committee (National Institute on Drug Abuse, Intramural Research Program, Baltimore, MD), approval ID 07-CNRB-61. Young adult (6–8 weeks) male heterozygous Nurr1 mice (Nurr1+/−), originally generated by Dr. Thomas Perlmann [1], and their littermate wild-type controls (+/+), were bred at NIDA. All animals were genotyped as previously described [1]. Animals were separated into 3 groups. (A) Single binged group (1XMETH): Animals received saline injections at 6–8 weeks old and received binge injections (10 mg/kg, x4, every 2 hours, s.c.) 5 months later. (B) Double binged group (2XMETH): Animals received binge injections (10 mg/kg, x4, every 2 hours, s.c.) at 6–8 weeks old and received a second binge (10 mg/kg, x4, every 2 hours, s.c.) 5 month later. (C) Control group: Animals received saline injections (0.01 ml/10 g, x4, every 2 hours, s.c.) at 6–8 weeks old, repeated 5 months later. During the injection period, animals were housed individually without bedding.

### TH immunostaining

Animals were anesthetized and perfused transcardially with saline followed by 4% paraformaldehyde (PFA) in phosphate buffer (PB; 0.1 M; pH 7.2) on the 4^th^ day after METH injections. The brains were dissected, postfixed in PFA for 16 hours, and transferred to 18% sucrose in 0.1 M PB for at least 16 hours. Serial sections of the entire brain were cut at 25 µm thickness in a cryostat. One series from every 4th section was stained for each antibody used. In order to control for staining variability, specimens from all experimental groups were included in every batch and reacted together in a net well tray under the same conditions. Sections were rinsed in 0.1 M phosphate buffer, and blocked with 4% bovine serum albumin (BSA) and 0.3% Triton x-100 in 0.1 M PB. Sections were then incubated in a primary antibody solution rabbit anti-TH (Chemicon, Temecula, CA) or rat anti-DAT (Chemicon, Temecula, CA) diluted (1∶500) in 4% BSA and 0.3% Triton x-100 in 0.1 M PB for 24 hours at 4°C. Sections were rinsed in 0.1 M PB and incubated in biotinylated goat anti-rabbit IgG for TH or anti-rat IgG for DAT in the buffer (1∶200; Vector Laboratories, Burlingame CA) for 1 hour, followed by incubation for 1 hour with avidin-biotin-horseradish peroxidase complex. Staining was developed with 2,3′ diaminobenzidine tetrahydrochloride (0.5 mg/mL in 50 mM Tris-HCl buffer 7.4). Control sections were incubated without primary antibody. Sections were mounted on slides, and cover slipped. Histological images were acquired using an Infinity3 camera and NIKON 80i microscope. TH and DAT immunoreactivity in striatum was visualized with the use of a Nikon super-coolscan 9000 scanner.

The optical density of TH and DAT immunoreactivity in striatum was analyzed using Scion Image (ver 4.02) and averaged from 3 sections with a visualized anterior commissure (AP: +0.26 mm, +0.14 mm, +0.02 mm to bregma). TH fiber optical density in substantia nigra pars reticulata (SNpr) and TH neuronal density in substantia nigra pars compacta (SNpc) were quantified by Nikon NIS-Elements software and averaged from 3 sections (AP:−3.28 mm, −3.40 mm, −3.52 mm to bregma). TH optical density and TH neuron counts from right and left hemispheres were averaged in each mouse for statistical analysis. All immunohistochemical measurements were done by blinded observers.

### Quantitative reverse transcription-PCR (qRT-PCR)

Mice were euthanized and the brains were immediately harvested and chilled on ice. The SN was dissected out and total RNA was extracted following the instructions from the manufacturer (RNAqueous, Ambion). Total RNA (1 µg) was treated with RQ-1 Rnase-free Dnase I and reverse transcribed into cDNA using random hexamers by AMV reverse transcriptase (Roche). cDNA levels for HPRT1 (hypoxanthine phosphoribosyltransferase 1), Hmbs (hydroxymethylbilane synthase) and Nurr1 were determined using specific universal probe Library primer probe sets (Roche) by quantitative RT-PCR. For each sample, duplicates were measured in real-time PCR and the results were repeated at least once with similar results. Primers and FAM-labeled probes used in the quantitative RT-PCR for each gene are as follows:

HPRT: forward primer (5′ –tgatagatccattcctatgactgtaga);reverse primer (5′ –aagacattctttccagttaaagttgag);probe (mouse universal probeLibrary probe #22, Roche)
Hmbs : forward primer (5′ – tccctgaaggatgtgcctac);reverse primer (5′-acaagggttttcccgtttg);probe (mouse universal probeLibrary probe #79, Roche)
Nurr1 : forward primer (5′ – tcagagcccacgtcgatt);reverse primer (5′- tagtcagggtttgcctggaa);probe (mouse universal probeLibrary probe #64, Roche)

### In vivo electrochemistry

DA clearance in striatum was measured at 3 days after METH or saline injections. Animals were anesthetized using urethane (1.25 g/kg, i.p.). In-vivo chronoamperometric measurements of extracellular dopamine (DA) concentration were performed as previously described (Zhou et al. 1996). The recordings were taken at rates of 10 Hz continuously using Nafion-coated carbon-fiber working electrodes (tip = 30 µm; SF1A, Quanteon, Lexington, Kentucky) and a microcomputer-controlled apparatus (FAST system, Quanteon). The clearance of DA was measured by changes in extracellular DA concentration after microinjection of DA into the striatal parenchyma (AP:0–0.5 mm and Lat 1.5 mm from Bregma, DV 1.5–3.5 mm from brain surface). DA (<20 pmole in 100 uM solution) was locally applied through a micropipette. The working electrode and the micropipette were mounted together with sticky wax; tips were separated by 150 µm. The electrode/pipette assembly was lowered into striatum (AP 0–0.5 mm, M/L 2.0 mm relative to bregma and 1.5 to 3.5 mm below the dura). Local application of DA from the micropipette was performed by pressure ejection using a pneumatic pump (BH2, Medical System). The ejected volume was monitored by recording the change in the fluid meniscus in the pipette before and after ejection using a dissection microscope.

### Stereologic Analysis

Unbiased stereological counts of TH-positive (TH+) neurons within the substantia nigra pas compacta (SNpc) were performed using stereological principles and analyzed with StereoInvestigator software (Microbrightfield, Williston, VT). Optical fractionator sampling [16] was carried out on a Leica DM5000B microscope (Leica Microsystems, Bannockburn, IL) equipped with a motorized stage and Lucivid attachment (40X objective). Midbrain dopaminergic groups were outlined on the basis of TH immunolabeling, with reference to a coronal atlas of the mouse brain (Franklin and Paxinos, [17]). For each tissue section analyzed, section thickness was assessed in each sampling site and guard zones of 2.5 µm were used at the top and bottom of each section. Pilot studies were used to determine suitable counting frame and sampling grid dimensions prior to counting. The following stereolgic parameters were used in the final study: grid size, (X) 220 µm, (Y) 166 µm; Counting frame, (X) 68.2 µm, (Y) 75 µm, depth was 20 µm. Gundersen coefficients of error for m = 1 were all less than 0.10. Stereologic estimations were performed with the same parameters in the SNpc of wildtype or Nurr1 heterozygous mice that received saline, 1XMETH or 2XMETH treatment (n = 6) for each treatment group.

## Results

### Nurr1 mRNA levels are decreased in heterozygous (+/−) mice

**Figure 1:**
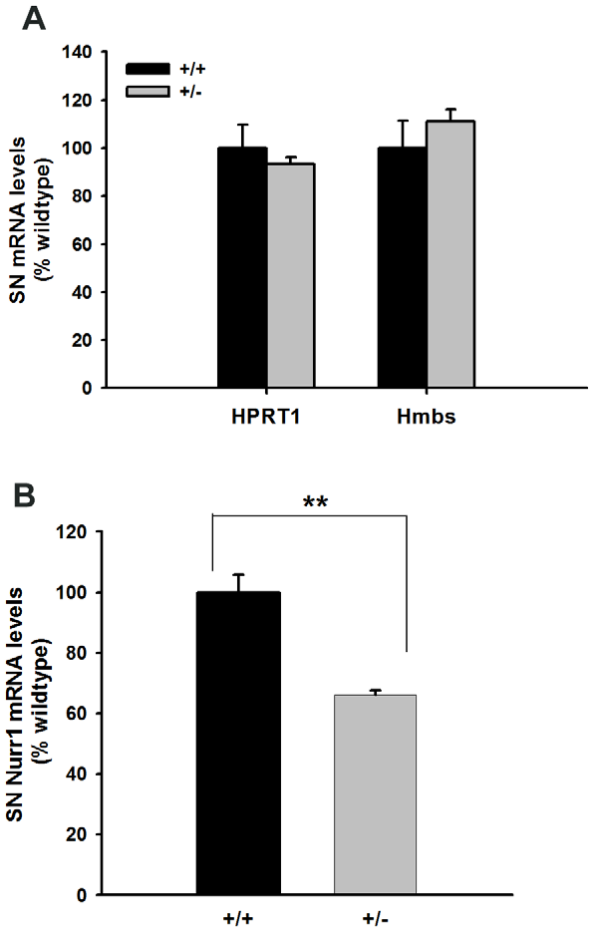
Nurr1 mRNA levels are decreased in the brains of Nurr1 heterozygous mice. Substantia Nigra (SN) mRNA levels for Nurr1, HPRT1 and Hmbs were measured in adult Nurr1 +/+ (n = 5) and +/− mice (n = 5) by qRT-PCR. Two housekeeping genes (HPRT1 and Hmbs) did not differ between +/+ and +/− mice (panel A). For each sample, Nurr1 mRNA levels were normalized to the housekeeping gene Hmbs mRNA levels. Nurr1 mRNA levels are significantly decreased (66% of wildtype) in +/− mice compared to levels in +/+ mice (**p<0.005, Student's t-test).

We measured Nurr1 and housekeeping genes HPRT1 and Hmbs mRNA levels in substantia nigra of Nurr1 +/− and +/+ mice using quantitative real-time RT-PCR. There was no difference between the +/− (n = 5) and +/+ (n = 5) for both housekeeping genes HPRT1 and Hmbs (Figure 1A). In contrast, there was a 34% reduction of Nurr1 mRNA levels in Nurr1 +/−, compared to +/+ mice (p<0.005, Student's t-test). These data suggest that the expression of Nurr1 was significantly reduced in +/− mice.

### Loss of striatal TH immunoreactivity (THir) after single or double binge METH injection

**Figure 2:**
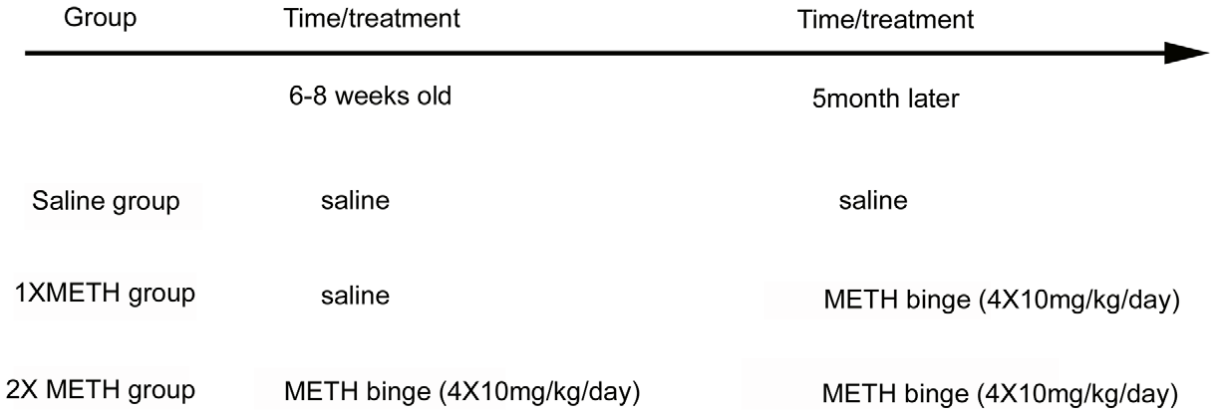
Treatment groups and schedules. Each group contained both Nurr1 +/+ and +/− mice. Saline group mice received saline injections when they were 6–8 weeks old and 5 months later. 1XMETH group mice received saline injections when they were 6–8 weeks old and received 1XMETH binge treatment (4X10 mg/kg) 5 months later. 2XMETH group mice received METH binge treatment (4X10 mg/kg) at both time points.

**Figure 3:**
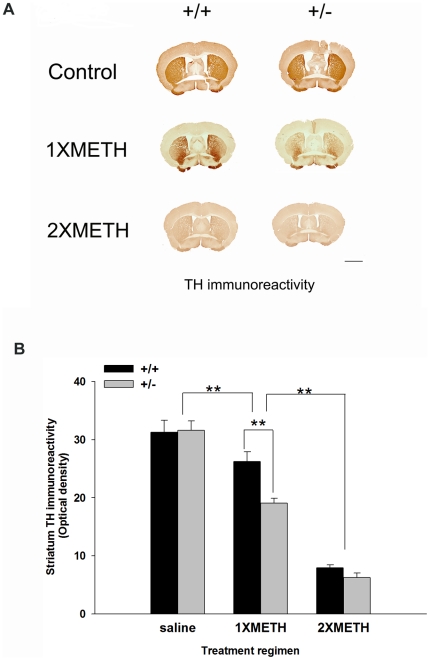
Nurr1 +/− mice exhibit greater reduction of striatal TH-immunoreactivity following METH exposure. (A) METH injection reduced THir in +/+ and +/− mice following one time binge METH exposure (1X METH) and repeated binge METH exposure (2XMETH). Calibration = 2 mm. (B) Quantitative THir fiber density analysis indicated that binge METH treatment significantly reduces THir in striatum and 2XMETH induced a further reduction of the THir in both +/+ and +/− mice. There is a significant difference between saline and 1XMETH group (p<0.001, Two-way ANOVA) and between 1XMETH and 2XMETH group (p<0.001, Two-way ANOVA) in striatal THir. In addition, there is also a significant difference (p<0.001) between +/+ and +/− mice after 1XMETH injection.

Animals were subjected to saline, a single binge (1XMETH) or double binge exposure (2XMETH) as illustrated in Fig 2. A total of 40 mice were sacrificed on the 4^th^ day after the last METH or saline injection. No difference in THir (optical density) was found between Nurr1 +/− (n = 6) and +/+ (n = 6) mice after saline injection (Fig 3A, B saline group, p = 0.858 Two-way ANOVA, post hoc Newman-Keuls test). 1XMETH significantly reduced THir density in striatum in both Nurr1 +/+ (n = 6) and +/− mice (n = 6) (p<0.001, Two-way ANOVA, post hoc Newman-Keuls test); however, the loss of THir optical density was more prominent in Nurr1 +/− mice (p<0.001, Two-way ANOVA, post hoc Newman-Keuls test) suggesting an increased vulnerability in these animals. Moreover, animals receiving double, comparing to single, METH binge showed a much greater loss of THir in striatum (Fig. 3A,B, p<0.001, Two-way ANOVA, post hoc Newman-Keuls test). There was no difference between the +/+ (n = 8) and +/− (n = 8) animals after 2XMETH; both genotypes lost about 70% of the THir in striatum (p = 0,317, Two-way ANOVA, post hoc Newman-Keuls test). In summary, we found that METH treatment significantly decreases THir in striatum (Two-way ANOVA, treatment, F_2, 77_ = 189.410, p<0.001) and Nurr1 +/− showed a significantly exacerbated loss in THir in stratium (Two-way ANOVA, genotype, F_2, 77_ = 6.771, p = 0.011). There is a significant interaction between the treatment and genotype (Two-way ANOVA, genotype x treatment, F_2,77_ = 3.916, p = 0.024).

### Loss of SNpr THir fibers after single or double binge METH injection

**Figure 4:**
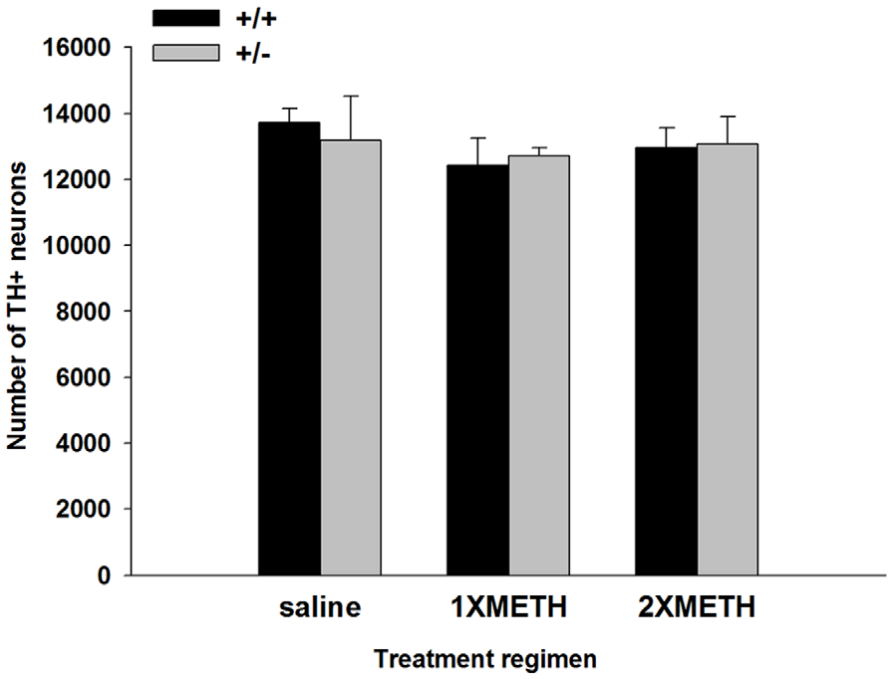
Stereological counts of SNpc TH-positive DAergic neuron numbers. DAergic neuron numbers in Nurr1 +/+ and +/− mice treated with saline, 1XMETH and 2XMETH were estimated using unbiased stereologic principles. METH binge treatment does not lead to loss of dopaminergic neurons in SNpc (p = 0.556, F_(2,17)_ = 0.617, Two-way ANOVA). Furthermore, there is no difference between wt and heterozygous mice (p = 0.957, F_(1,17)_ = 0.003, Two-way ANOVA).

**Figure 5:**
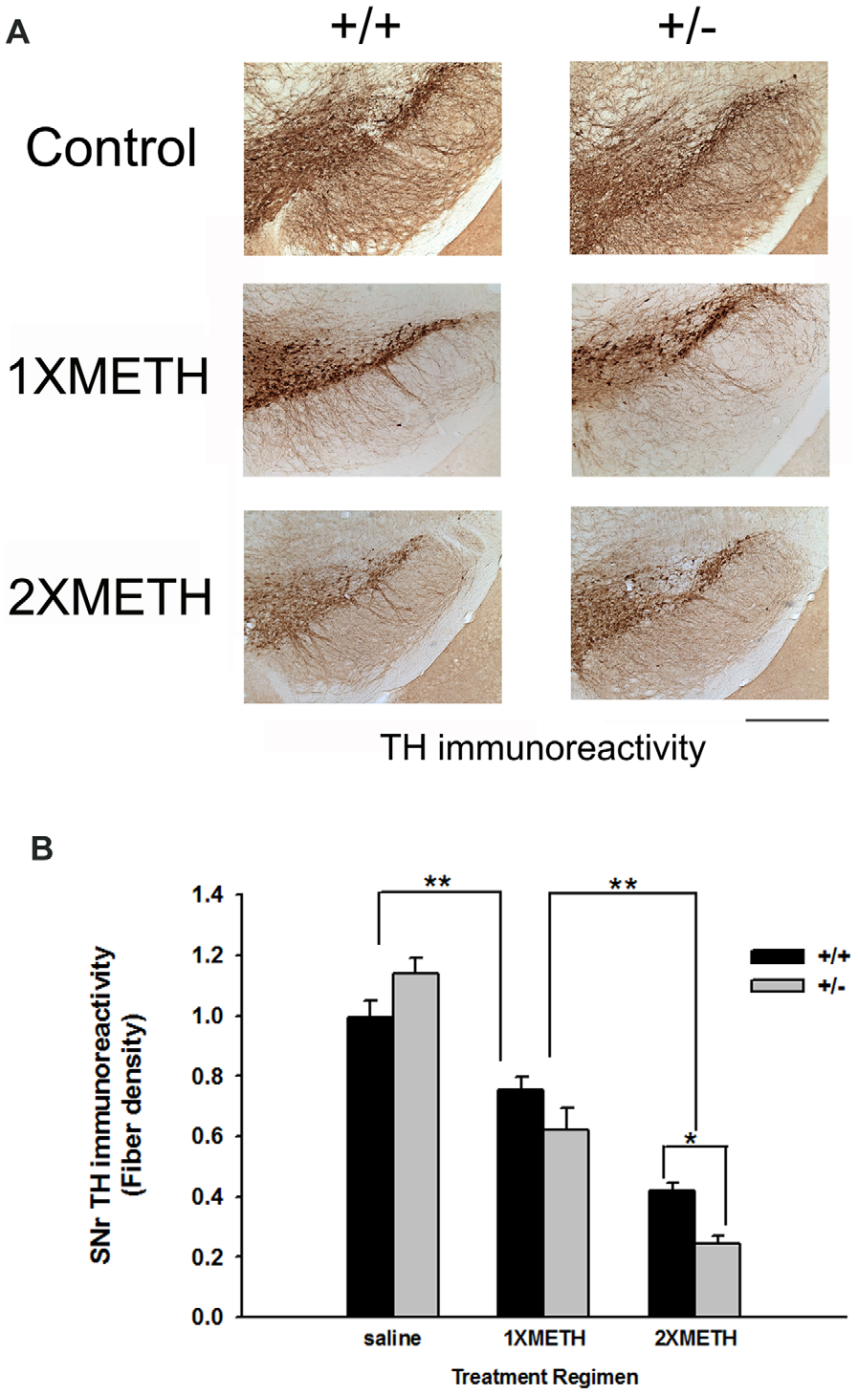
Nurr1 +/− mice exhibit decreased TH-immunoreactivity in SNpr following methamphetamine exposure. (A) TH immunostaining indicates that METH administration augments the reduction in THir in the SNpr of Nurr1 +/−, compared to +/+ mice following repeated METH binge treatment. Calibration bar = 500 um. (B) THir fiber density analysis indicated METH treatment (1xMETH) reduced THir fiber density in SNpr in +/+ and +/− mice (p<0.001, Two-way ANOVA) and repeated METH binge treatment (2XMETH) further reduced THir fiber density in SNpr (p<0.001, Two-way ANOVA). A trend of greater reduction in TH immunoreactivity was found in the Nurr1 +/− mice following 1XMETH (p = 0.058, Two-way ANOVA, post hoc Newman-Keuls test) and a significant decrease in THir in SNpr was found in Nurr1 +/− mice following repeated METH treatment (2XMETH). (p<0.05, Two-way ANOVA, post hoc Newman-Keuls test).

We next examined the effect of METH binge exposure on the substantia nigra (SN). To evaluate DAergic neuron number in SN, we performed unbiased stereological counts in various animal groups. This unbiased method of cell counting is not affected by either the volume of reference (SNpc) or the size of the counted elements (neurons) [16]. The results showed that decreased level of Nurr1 in heterozygous mice did not affect the number of dopaminergic neurons in adult control mice (saline group, wt  = 13714; het  = 13194, p = 0.957, F_(1,17)_ = 0.003, Two-way ANOVA). Furthermore, neither 1XMETH nor 2XMETH led to a decrease in the number of TH-positive neurons in SNpc in both genotypes (p = 0.556, F_(2,17)_ = 0.617, Two-way ANOVA; Fig 4). TH fiber density in SNpr was analyzed using fiber density of THir. 1XMETH significantly reduced density of TH fibers in SNpr (Two-way ANOVA, treatment p<0.001, F_(2,48)_ = 98.444; p<0.001, post hoc Newman-Keuls test; Fig 5A, B) in both Nurr1 +/+ (n = 10) and +/− (n = 10) mice; Nurr1 +/− mice showed a trend towards greater decrease in THir in SNpr (Two-way ANOVA, p = 0.058, post hoc Newman-Keuls test). 2XMETH caused a further reduction in TH fiber density in SNpr in both Nurr1 +/+ (n = 8) and +/− (n = 7) animals (Two-way ANOVA, p<0.001, post hoc Newman-Keuls test). Moreover, 2XMETH caused a greater reduction of TH immunoreactivity in SNpr in Nurr1 +/−, compared to +/+ mice (Two-way ANOVA, p = 0.028, post hoc Newman-Keuls test, Fig 5A, B). In summary, METH treatment significantly decreases THir in SNpr (Two-way ANOVA, treatment, F_2, 48_ = 98.444, p<0.001) and there is a significant treatment-dependent difference between +/− and +/+ mice (Two-way ANOVA, genotype x treatment, F_2,48_ = 5.692, p = 0.006). Taken together, these histological findings suggest that Nurr1 +/− mice are more sensitive to METH –mediated damage in DAergic neurites.

### Electrochemical measurement of dopamine clearance in Nurr1 +/+ and +/− mice after single or double binge METH injection

**Figure 6:**
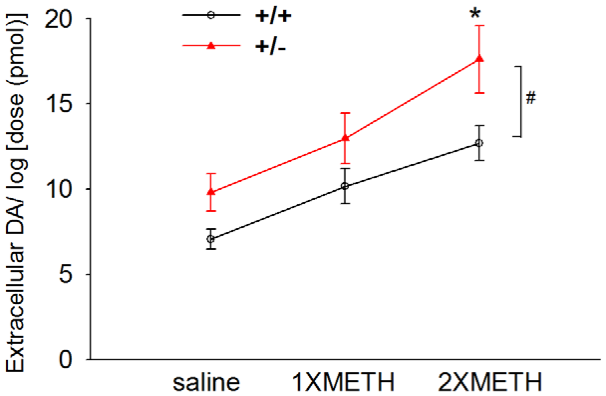
Further reduction of DA clearance in the striatum after repeated METH binge in Nurr-1 +/− mice. Extracellular DA concentration was measured using chronoamperometry after local application of low doses (10.2+/−0.6 pmole/site) of DA through micropipettes in mouse striatum after saline, 1xMETH or 2XMETH treatment. Amplitude of DA signals was normalized by comparison to the log of dose used. Extracellular DA concentration was significantly enhanced in Nurr1 +/−, compared to +/+ mice, after 2XMETH treatment (*, p<0.05, Two-way ANOVA, post hoc Newman-Keuls test).

DA clearance was examined after local application into the striatum in 23 mice at 3 days after the last injection of METH or saline. Extracellular DA levels were recorded in 136 striatal sites between 1.5 mm to 3.5 mm below the brain surface. Of these, 72 sites were taken from Nurr1 +/− mice while 64 sites were recorded from wt (+/+) mice. Local application of DA (10.2 +/− 0.6 pmole/site) induced an increase in extracellular DA levels. Previous chronoamperometric studies have shown a dose-dependant relationship between the peak of extracellular DA and log dose of applied DA through a micropipette in rat striatum [18]. In this study, the amplitude of DA release was also normalized by comparison to the log of dose used (in pmole). We found that extracellular DA concentration was significantly enhanced after local DA application in Nurr1 +/− mice (F_1,145_ = 13.488, p<0.001, two way ANOVA). A significant decrease in clearance was found in Nurr+/− mice after 2XMETH treatment (Fig 6, p = 0.010, post-hoc Newman-Keuls test). No significant difference was found after 1XMETH (p = 0.126) or saline treatment (p = 0.118). These data suggest that 2XMETH reduced DA clearance in Nurr1 +/− mice.

### Examination of striatal DAT immunoreactivity (DATir) after single or double binge METH injection

**Figure 7:**
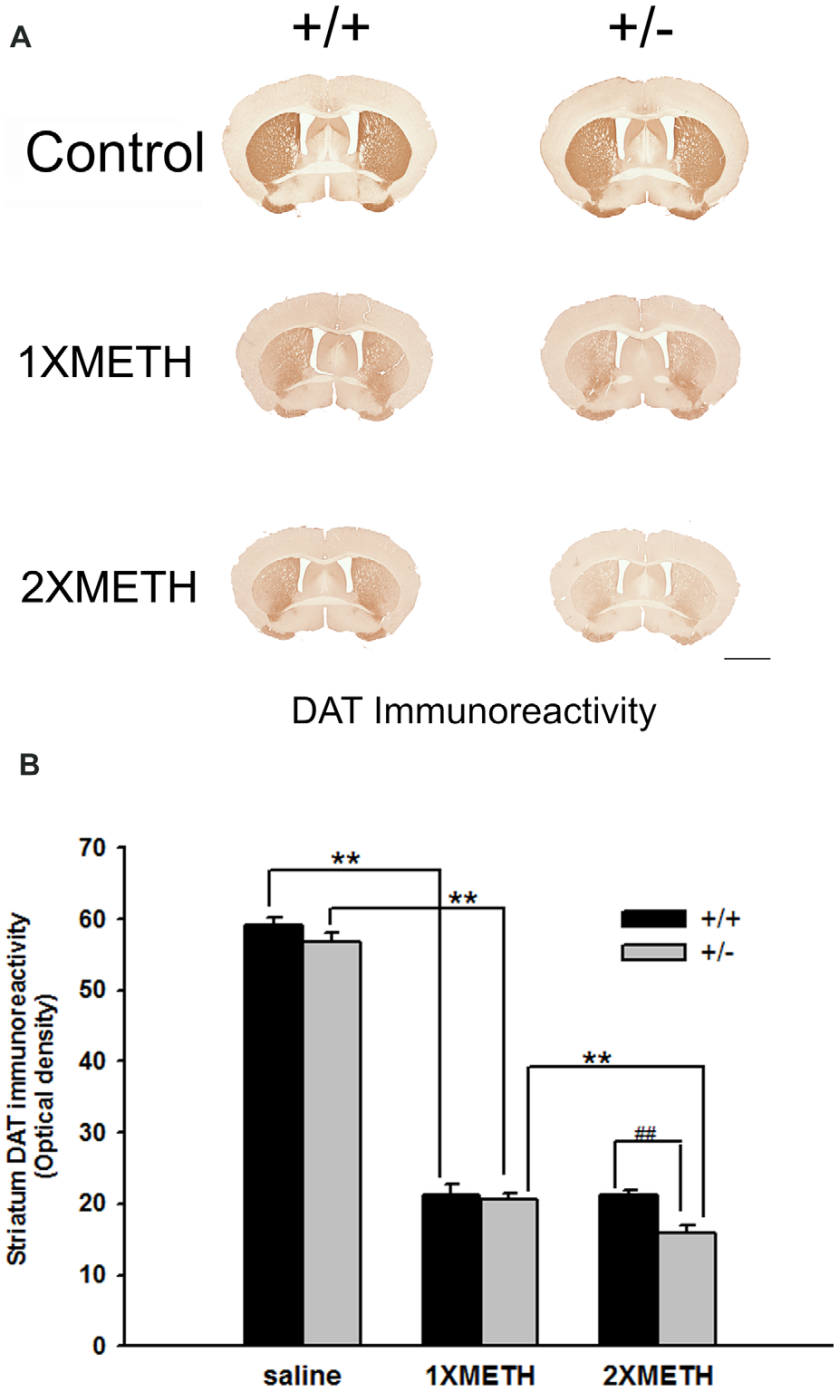
Decreased DA clearance due to loss of DAT expression in striatum. (A) DAT immunostaining indicates that METH administration augments the reduction in DATir in the striatum of Nurr1 +/−, compared to +/+ mice following repeated METH binge treatment. Calibration bar = 2 mm. (B) DATir fiber density analysis indicated METH treatment (1xMETH) reduced DATir fiber density in striatum in +/+ and +/− mice (p<0.001, Two-way ANOVA) and repeated METH binge treatment (2XMETH) further reduced DATir fiber density in striatum in Nurr1 +/− mice compared to +/+ mice (p<0.001, Two-way ANOVA). Nurr1 +/− mice showed decreased DATir after 2X METH treatment compared to +/+ animals, but not in saline-treated or 1XMETH-treated groups, a result consistent with decreased DA clearance in 2XMETH-treated Nurr1 +/− mice. ** indicates that there is a significant difference between the treatment (p<0.001, Two-way ANOVA) and ## indicates that there is significant difference between the genotypes after 2XMETH treatment (p<0.001, Two-way ANOVA).

To examine whether the reduced DA reuptake is due to loss of DAT protein in striatum, we examined the level of DATir by optical density in striatum in saline and METH -exposed (+/+) and (+/−) animals. No difference in DATir (optical density) was found between Nurr1 +/− (n = 6) and +/+ (n = 6) mice after saline injection (Fig 7A, B saline group, p = 0.127, Two-way ANOVA, post hoc Newman-Keuls test). Consistent with the in vivo electrochemistry data, we found that 1XMETH significantly reduced DATir in both Nurr1 +/+ (n = 6) and +/− mice (n = 6) (Fig 7A, Two-way ANOVA, p<0.001, post hoc Newman-Keuls test). No significant difference was found between these two genotypes (Fig 7B, panel 2, Two-way ANOVA, p = 0.681, post hoc Newman-Keuls test). In contrast, following 2XMETH, there was a significant reduction of DAT optical density in Nurr1 +/− (n = 10), compared to +/+ mice (n = 10) (Fig 7A, B, Two-way ANOVA, p<0.001, post hoc Newman-Keuls test Statistics). In summary, we found that METH treatment significantly decreases the DATir in striatum (Two-way ANOVA, treatment, F_2, 82_ = 882.560, p<0.001) and Nurr1 +/− showed a significant exacerbated loss in DATir in striatum after 2XMETH (Two-way ANOVA, p<0.001). There is a significant interaction between the treatment and genotype (Two-way ANOVA, genotype x treatment, F_2, 82_ = 4.483, p = 0.014). Taken together, the immunohistochemical measurements on DAT density correlate well with the electrochemistry data on DA clearance and suggests that the decreased DA clearance in Nurr1 (+/−) mice after 2XMETH could be due to an elevated loss of DAT protein in the neuronal terminals.

## Discussion

Previous studies have indicated that the nigrostriatal DA pathway is vulnerable to the toxic effect of METH. High doses of METH increase extracellular concentration of DA, DA metabolites, and glutamate in the nigrostriatal terminal fields *in vivo* [19], [20]. These toxic metabolites/compounds may contribute to the acute toxicity of METH at the DAergic terminals. In this study, we demonstrated that repeated exposure to high doses of METH caused further neurodegeneration in the nigrostriatal DAergic pathway. We found that METH binge, given at early life (week 6–8 in mice), potentiated the reduction of TH and DAT immunoreactivity in striatum and nigra after a second METH binge, given at 5 month later, a long time effect which has not been reported previously. These data also suggest that after the initial METH binge, subsequent adult exposure increases METH toxicity.

We also demonstrated the interaction of METH and Nurr1 in the nigrostriatal DAergic pathway. There is a further reduction of TH or DAT as well as DA clearance in Nurr-1 (+/−) mice after repeated treatment with high doses of METH, suggesting a synergistic neurodegenerative response between deficiency in Nurr1 and repeated METH exposure in DAergic neurons. The biological mechanisms for this interaction have not been clearly identified. It has been shown that Nurr1 +/− mice had an elevated nNOS and nitration in striatum [15]. Deficiency in Nurr1 has also been suggested to play a role in the induction of apoptosomes via NO. Furthermore, Nurr1+/− mice had an elevated p53 protein level as well as an increased Cytochrome C levels in the striatum after a single METH binge treatment [15]. All these toxic responses may result in the greater loss of THir and DATir in these animals as shown in our study. Since DA clearance and DAT expression in striatum were reduced in +/− mice after repeated METH binge treatment, it is also possible that the overflow of DA in these animals further potentiates damages to DAergic neurons after METH treatment. Decreased DA clearance might also account for the elevated locomotor activity observed in Nurr1 +/− mice compared to +/+ after METH administration as previously reported by our group [21].

A recent study from Cadet et al [22] demonstrated a preconditioning protective effect of METH on subsequent METH binge exposure if the pretreatment of METH was given at lower doses over a longer period of time. The differential effects of METH pretreatment on the subsequent exposure might be due to different regimens of drug administration. While chronic low dose pretreatment could induce certain protective genes such as SOD (Superoxide Dismutase) and trophic factors [22], high dose METH binge treatment leads to downregulation of trophic factors [23] and anti-apoptotic genes such as bcl-2 [15]. In contrast to the protective effect of low dose chronic preconditioning, our results suggest that intermittent binge exposure causes detrimental instead of protective effects on subsequent METH exposure. To further define mechanisms on how difference in Nurr1 expression affects methamphetamine vulnerability, given data from Cadets lab [22] on apoptosis and trophic factor changes, we also utilized RTPCR to study expression of apoptotic genes (Bcl2/BAX) as well as trophic factors (BDNF, SHH). We found no significant changes between groups, suggesting other mechanisms may be involved (data not shown). Therefore, the elevated vulnerability of Nurr1 +/− mice to repeated METH treatment is at least not mediated by the Bcl2/Bax pathway. This speculation is consistent with our results showing that there is no actual DAergic cell loss in SNpc after METH binge treatment.

Previous studies have demonstrated that the suppression of KCl-mediated DA release is reversible 1 to 6 months after high dose METH administration in adult Fisher rats [24], suggesting a transient suppression of certain DAergic phenotypes and spontaneous recovery of DA release function after acute METH treatment in rodents. Consistent with previous reports, using unbiased stereological counts of SNpc DAergic neuron number, we did not observe a loss of dopaminergic neurons in SNpc in METH binge treated animals. Most of the acute toxicity observed, including suppressed locomotor activity, loss of THir fibers in striatum and loss of THir projections in SNr, recover in 2–4 weeks after the METH binge exposure (data not shown). However, we also found a long term neurodegenerative effect in DAergic neuronal terminals after METH binge treatment in animals with a deficiency in Nurr1 expression. Since Nurr1 is essential for the survival and function of doapminergic neurons [5], it is possible that Nurr1 may be involved in the spontaneous recovery after METH exposure. Deficiency in Nurr1 expression levels in +/− mice may lead to a compromised functional recovery. Small molecules that could upregulate Nurr1 activity might thus be a therapeutic target for METH toxicity and PD.

It has been shown that newborn Nurr1 +/− mice have significantly reduced levels of Nurr1 protein and DA in the striatum [1], indicating that nigrostriatal DA levels are affected by Nurr1 mRNA dosage. Moreover, in adulthood, these heterozygous mice showed increased vulnerability to the neurotoxin MPTP (1-methyl-4-phenyl-1,2,3,6-tetrahydropyridine) compared to their wildtype littermates [6]. Similarly, DAergic trophic factors have been reported to alter DAergic function after METH exposure. It has been reported that deficiency in GDNF (Glial cell line-derived neurotrophic factor) level leads to a greater vulnerability to METH binge exposure in GDNF +/− mice [25]. We have also reported that reduced BMP7 (Bone morphogenetic protein 7) levels in BMP7 +/− mice caused a larger adverse effect by METH binge treatment [23]. Whether there is a common mechanism for these trophic factors in protecting DA neurons from METH exposure requires further investigation.

In summary, early METH binge exposure in young adulthood leads to long term effects in the nigrostriatal system and results in a more marked dysfunction when animals are exposed to METH again later in life. This adverse effect is further exacerbated in mice that have a decreased level of Nurr1. Therefore, lowered Nurr1 levels may predispose individuals to greater acute and/or long term toxicity of METH in the nervous system. Our data suggest that METH binge exposure in early life can exacerbate neurodegeneration induced by a 2^nd^ METH exposure, especially in Nurr-1 deficient animals. It is possible that mutations or polymorphisms in the Nurr1 gene in humans, which lead to lower levels of Nurr1 expression, may predispose certain individuals to greater susceptibility to neuronal disorders or greater susceptibility to neurotoxicants after repeated exposure. Small molecules that can regulate Nurr1 function and activity might be a candidate medications development target for METH toxicity and PD.
